# Stimulus expectation alters decision criterion but not sensory signal in perceptual decision making

**DOI:** 10.1038/s41598-017-16885-2

**Published:** 2017-12-06

**Authors:** Ji Won Bang, Dobromir Rahnev

**Affiliations:** School of Psychology, Georgia Institute of Technology, Atlanta, GA 30332 USA

## Abstract

Humans are more likely to report perceiving an expected than an unexpected stimulus. Influential theories have proposed that this bias arises from expectation altering the sensory signal. However, the effects of expectation can also be due to decisional criterion shifts independent of any sensory changes. In order to adjudicate between these two possibilities, we compared the behavioral effects of pre-stimulus cues (pre cues; can influence both sensory signal and decision processes) and post-stimulus cues (post cues; can only influence decision processes). Subjects judged the average orientation of a series of Gabor patches. Surprisingly, we found that post cues had a larger effect on response bias (criterion *c*) than pre cues. Further, pre and post cues did not differ in their effects on stimulus sensitivity (*d’*) or the pattern of temporal or feature processing. Indeed, reverse correlation analyses showed no difference in the temporal or feature-based use of information between pre and post cues. Overall, post cues produced all of the behavioral modulations observed as a result of pre cues. These findings show that pre and post cues affect the decision through the same mechanisms and suggest that stimulus expectation alters the decision criterion but not the sensory signal itself.

## Introduction

When given an expectation about a forthcoming stimulus, human subjects report the expected stimulus with greater frequency^1–5^. What processes underlie this expectation effect? Two broad alternatives have been proposed.

First, it is possible that stimulus expectation alters the sensory signal itself ^6–8^. According to this view, the stimulus is processed differently in the presence of expectation. This possibility is supported by popular theories such as predictive coding^9,10^ that postulate a process of top-down signals sent from downstream to upstream areas that affect how the forthcoming stimulus will be processed.

Second, it is possible that stimulus expectation alters the decision criterion^6,11^. According to this view, the sensory processing of the stimulus remains exactly the same but downstream areas change the response using the information from the cue. This possibility is tacitly assumed by more abstract theories such as Bayesian Decision Theory^12^ and Signal Detection Theory^13,14^ which typically treat stimulus expectation as affecting the decision rule rather than the stimulus distributions.

**Figure 1:**
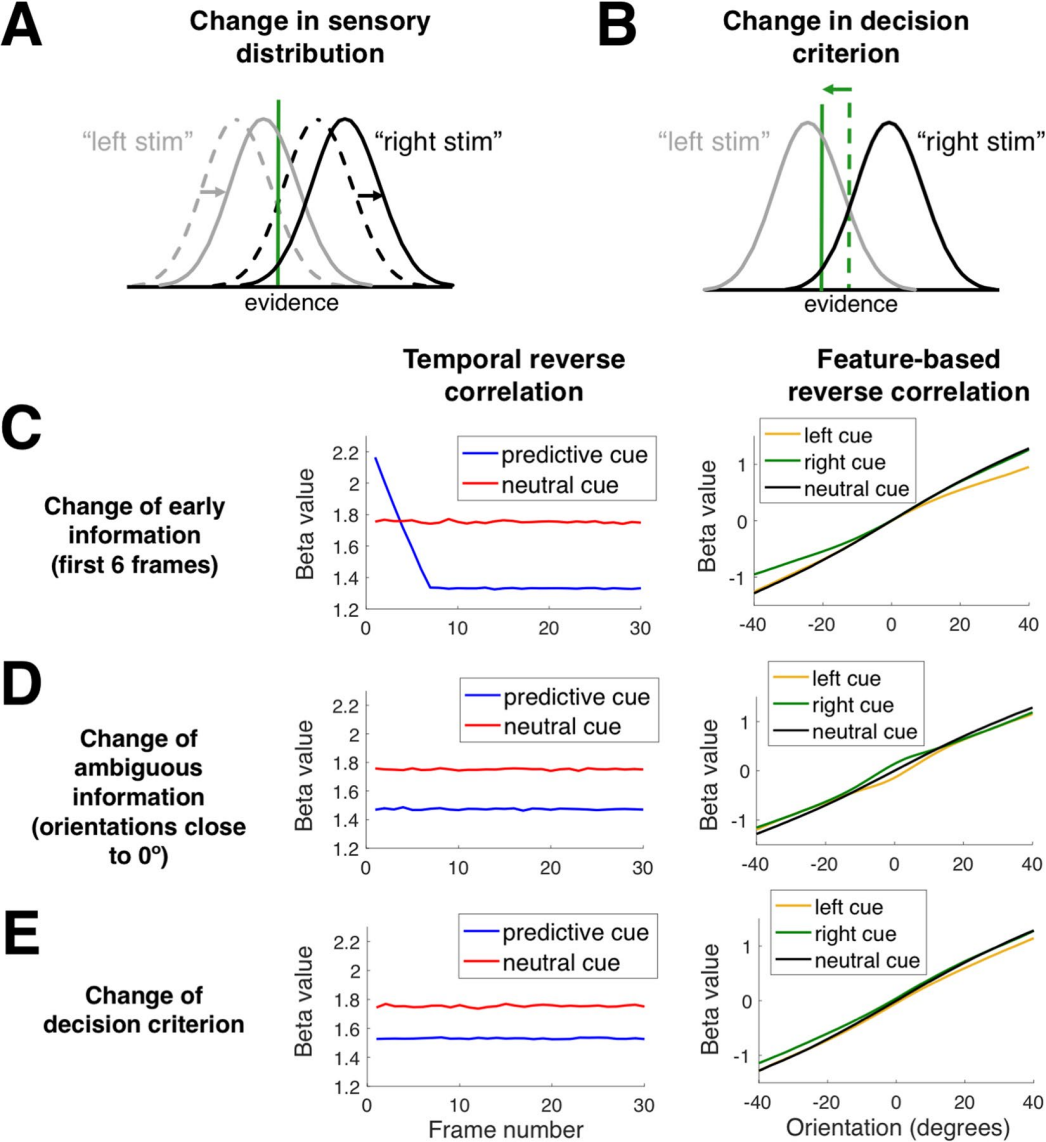
Possible effects of stimulus expectation. (**A**,**B**) A change of the pattern of subjects’ responses can be accommodated by Signal Detection Theory as either a shift in the sensory distributions (**A**) or a shift in the decision criterion (**B**). These two options are mathematically equivalent and therefore cannot be distinguished based on the pattern of subjects’ responses alone. (**C**–**E**) Depiction of putative influences of expectation on temporal and feature-based reverse correlation analyses. For temporal reverse correlations, we plot separately the information usage following predictive vs. neutral cues; for feature-based reverse correlations, we plot separately the information usage following left, right, and neutral cues. Expectation may bias the early sensory signal such that early Gabor patches (in this example, the first 6 patches) with cue-congruent orientations are over-weighted in the decision. Such an effect would lead to an L-shaped curve of temporal information usage (panel C, left), as well as strong under-weighting of cue-incongruent information (panel C, right). Alternatively, expectation may bias ambiguous sensory signal (e.g., it could bias orientations close to 0° toward the cued direction). Such an effect would lead to an overall underuse of sensory information for predictive cues (panel D, left), as well as a characteristic deflection of information usage for left and right cues around 0° (panel D, right). Finally, expectation may simply change the decision criterion, resulting in under-weighting of sensory information for predictive cues (panel E, left) and under-weighting of cue-incongruent information (panel E, right). Negative/positive orientations correspond to left/right stimuli.

The sensory signal and decision criterion accounts of expectation are difficult to distinguish based on standard behavioral data^15^. Indeed, if one only analyzes subjects’ choices and applies Signal Detection Theory, then expectation will manifest itself as a criterion change^13–15^. However, the reason for this change could be either a shift in the underlying sensory distributions with no change to the decision criterion (Fig. 1A), or a shift in the decision criterion with no change in the underlying sensory distributions (Fig. 1B). These two alternatives are mathematically equivalent and therefore cannot be distinguished without further manipulations.

The sensory signal and decision criterion accounts of expectation can, however, be adjudicated by varying the stimulus and employing reverse correlation analyses^16,17^. To demonstrate this point, we examined the results of reverse correlation analyses applied to simulated data generated via sensory signal or decision criterion changes. The simulations mirrored the parameters of our behavioral experiment, which presented a time-varying stimulus consisting of 30 sequential Gabor patches with orientations spanning from −45° to 45° (see Methods). In our simulations, reverse correlation revealed signatures of various putative expectation effects. For example, a change in early but not late sensory signal created a characteristic pattern of L-shaped temporal information usage for predictive cues (Fig. 1C), while a change in ambiguous but not unambiguous sensory signal created a characteristic central deflection in the pattern of feature-based information usage (Fig. 1D). On the other hand, a change in the decision criterion created its own pattern of temporal and feature-based information usage showing under-weighting of sensory information for predictive cues in temporal reverse correlation and of cue-incongruent information in feature-based reverse correlation (Fig. 1E).

These signatures of sensory signal vs. decision criterion changes can already be used to adjudicate between these two possibilities. However, the simulations above are based on the assumption that, except for the putative effects of expectation, subjects act as ideal observers. On the other hand, real subjects may not always treat sensory information in mathematically optimal manner^18–22^. Furthermore, subjects’ biases may interact with the putative effects of expectation thus making it hard to distinguish sensory signal from decision criterion effects of expectation.

We addressed this problem by employing post-stimulus cues. Standard expectation cues presented before the stimulus (pre cues) can potentially affect both the sensory signal and the decision criterion. On the other hand, if information from individual components of a time-varying stimulus is not available after stimulus offset, then post-stimulus cues (post cues) can only affect the decision criterion. To prevent sensory information related to individual Gabor patches from persisting into the post-stimulus period, we presented the Gabor patches for a single computer frame in the same spatial location (at fixation) so that they backward-mask each other. Indeed, backward masking ensures that reverberation of sensory information in sensory circuits is interrupted^23,24^. Note that in the absence of such backward masking effects, reverberations can continue for a few hundred milliseconds and post cues can retroactively act to enhance these persistent sensory signals^25^. In addition to the backward masking, we revealed the post-stimulus cue a substantial amount of time after the stimulus offset (500 ms). These design features ensured that our post cues could not affect the sensory signal and allowed us to test whether pre cues act through altering the sensory signal or decision criterion.

To anticipate, we found that post cues were surprisingly more effective in influencing subjects’ choices than pre cues. At the same time, pre and post cues did not differ in their effects on stimulus sensitivity, temporal or feature-based information usage. Finally, the reverse correlation analyses for both the pre and post cues showed the signatures of decision criterion changes. Thus, our results show that both pre and post cues influence subjects’ choices through the same mechanism and suggest that the influence of stimulus expectation is due to decision criterion rather than sensory signal changes.

## Results

**Figure 2:**
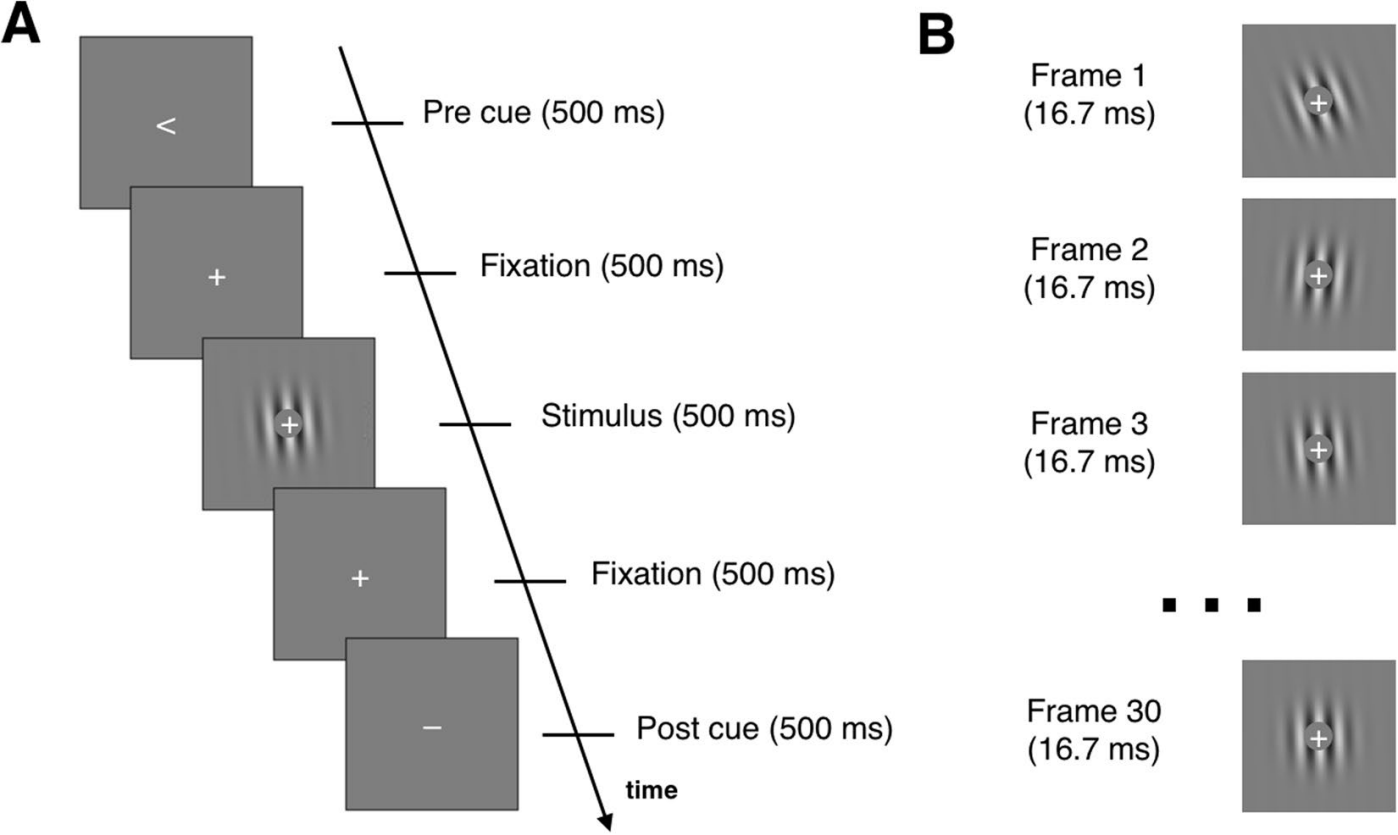
Task. (**A**) An example trial. The stimulus was preceded by a pre cue and followed by a post cue. Fixation periods were inserted between the cues and the stimulus. In alternating blocks, either pre or post cues were relevant (the other cue consisted of an uninformative horizontal line). The relevant cues were valid with 66.67% probability. On 25% of the trials, a neutral cue (consisting of a vertical line) was presented. The example trial above is drawn from a pre cue block. (**B**) The stimulus consisted of 30 Gabor patches with orientations drawn from a normal distribution determined for each subject using a staircase. Each Gabor patch was presented for one frame (16.7 ms).

We compared the influence of pre and post cues on subjects’ responses. The two types of cues were designed to be as equivalent as possible. Subjects performed left/right (i.e., counterclockwise/clockwise) discrimination judgments (Fig. 2A) on the average orientation of a series of 30 oriented Gabor patches (Fig. 2B). In alternating blocks, either a pre or a post cue with 66.67% validity was presented. To aid comparison, both the pre and post cue blocks included neutral (uninformative) cues.

### Post cues affect the criterion more than the pre cues

**Figure 3:**
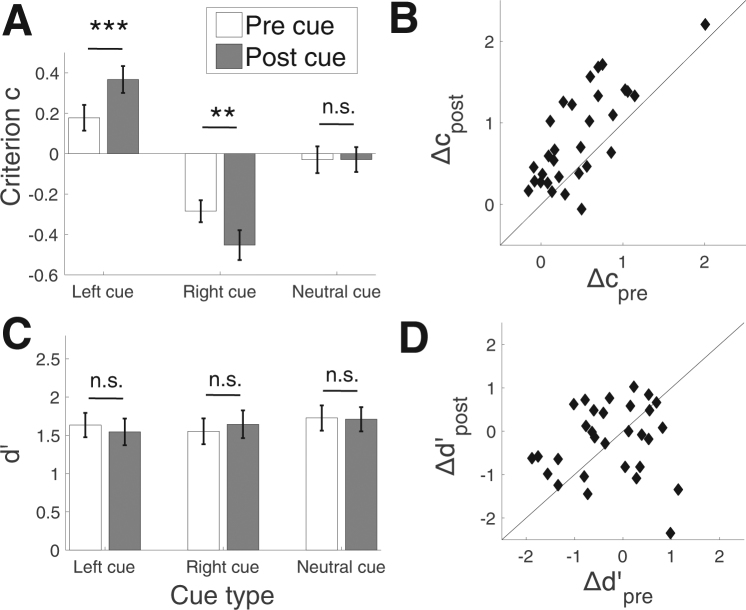
Behavioral effects. (**A**) Effect of pre and post cues on the decision criterion. Post cues had a larger effect on the criterion (larger bias away from zero) than pre cues. (**B**) Individual data for criterion effect. The criterion effect *Δc* was defined as the difference between the criterion for left and right cues. For the majority of subjects, this effect was larger for the post cues (points above the diagonal identity line). (**C**) Effect of pre and post cues on stimulus sensitivity *d’*. Pre and post cues did not differ in their effect on stimulus sensitivity. (**D**) Individual data for the *d’* effect. As with the criterion, the *d’* effect *Δd’* was defined as the difference in *d’* values between left and right cues. No difference in the effect was found for pre and post cues. Error bars show S.E.M. **p < 0.01, ***p < 0.001.

We first analyzed the influence of the cues on the response criterion. Left cues resulted in significantly positive criterion both when presented before (average *c* = 0.18; *t*(29) = 2.8, *p* = 0.009) and after (average *c* = 0.37; *t*(29) = 5.53, *p* = 0.000006) the stimulus (Fig. 3A), indicating a bias for “left” choices. Similarly, right cues resulted in significantly negative criterion both when presented before (average *c* = −0.28; *t*(29) = −5.21, *p* = 0.00001) and after (average *c* = −0.45; *t*(29) = −6.14, *p* = 0.000001) the stimulus, indicating a bias for “right” choices. Finally, neutral cues did not significantly bias the criterion for either cue type (pre cues: average *c* = −0.03; *t*(29) = −0.45, *p* = 0.65; post cues: average *c* = −0.03; *t*(29) = −0.48, *p* = 0.64).

Critically, when comparing pre and post cues directly, we found that post cues had a larger influence on the criterion (i.e., induced a larger deviation from *c* = 0) for both left (*t*(29) = 4.87, *p* = 0.00004) and right (*t*(29) = 2.9, *p* = 0.007) cues but not for neutral cues (*t*(29) = 0.01, *p* = 0.99).

In other words, even though they could only affect the decision criterion, post cues influenced the pattern of subject responses more than pre cues. This effect is especially evident when the criterion difference for left minus right cues is directly compared for pre and post cues (average *Δc*_*post*_ = 0.82, average *Δc*_*pre*_ = 0.46; *t*(29) = 5.07, *p* = 0.00002; Fig. 3B). The post cues had a larger effect on the criterion for 25 of our 30 subjects. It should be noted that, as in previous studies^1,5,13,26^, subjects adjusted their criterion less than optimal in response to both pre and post cues. Indeed, the optimal criterion shift between left and right cues across our pool of subjects was *Δc*_*optimal*_ = 1.13 (see Methods for how this value was computed), which is significantly larger than the criterion effect for post (*t*(29) = 2.91, *p* = 0.007) and pre (*t*(29) = 7.83, *p* = 1.2 * 10^−8^) cues.

### Inter-subject correlation between pre and post cue usage

As noted in Fig. 1A,B, the criterion effects observed above can be due to either sensory distribution or decision criterion shifts. Importantly, if pre cues only induced a sensory signal shift, then one may expect zero correlation between the criteria effects of pre and post cues (that is, no correlation between *Δc*_*pre*_ and *Δc*_*post*_). Indeed, post cues necessarily acted through a decision criterion shift and there is no *a priori* reason to assume that sensory signal shifts and decision criterion shifts within a subject will have comparable magnitudes.

Nevertheless, we observed a significant inter-subject correlation between the pre and post cue effects on criterion (*r* = 0.75, *p* = 0.000002). This result indicates that at least 56% of the inter-subject variability of the pre cue criterion effect can be explained by the inter-subject variability in the post cue criterion effect. However, the correlation between the two effects is limited by the reliability with which each effect can be computed^27^. We estimated the reliability of each effect by re-computing each measure from the odd vs. even trials and applying the Spearman-Brown formula^27^ to correct for the fact that we used only half of the trials in each case (see Methods for details). We found that the maximum expected correlation between pre and post cues, given the reliability with which we estimated them, is *r* = 0.82. This value is only slightly greater than the observed correlation of *r* = 0.75 and can be attributed to some subjects adopting slightly different decision criterion shift strategies for pre and post cues. These results suggest that, when taking the reliability of each measure into account, the correlation between the pre and post cue criterion effects is as high as one would expect had they been based on the same mechanisms.

### Pre and post cues have the same effect on stimulus sensitivity

While post cues had larger effects on the response criterion, pre and post cues did not differ significantly in their effect on stimulus sensitivity (left pre vs. left post cues: *t*(29) = 1.04, *p* = 0.31; right pre vs. right post cues: *t*(29) = −1.14, *p* = 0.26; neutral pre vs. neutral post cues: *t*(29) = 0.17, *p* = 0.87; Fig. 3C).

Note that despite the appearance of a possible interaction between left/right vs. pre/post cues, the *d’* difference between left and right cues did not differ significantly for pre and post cues (*t*(29) = 1.36, *p* = 0.19; Fig. 3D). Further, unlike for the response criterion, the effects on *d’* (for left minus right cues) did not correlate across subjects for pre and post cues (*r* = 0.08, *p* = 0.69).

### Pre and post cues have the same effect on temporal information usage

The analyses above suggested that pre cues act through the same mechanisms as post cues. However, as demonstrated in Fig. 1C–E, subtle effects of expectation are best captured through the use of reverse correlation analyses. These analyses can reveal how the stimuli were processed in both the temporal and feature domains.

**Figure 4:**
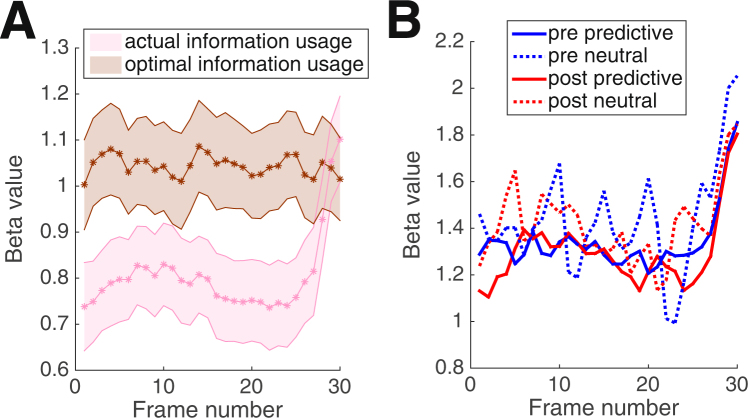
Temporal information usage. (**A**) The pattern of temporal information usage over the course of the 30 stimulus frames shows a pronounced recency effect such that the last two frames influenced the decision more. The optimal information usage is displayed for comparison. The line of optimal usage is not flat (even though optimally each frame should be weighted equally) since it was computed from noisy data. Shaded areas show S.E.M. (**B**) Temporal information usage for predictive and neutral cues did not differ by cue time (pre vs. post). The lines for neutral cues are noisier since they are based on fewer trials. All timecourses are smoothed for display purposes using a two-frame sliding window.

In the temporal domain, we investigated whether pre or post cues affected subjects’ information usage at any time throughout the 30 stimulus frames. We first combined all trials together and found that subjects’ information usage was relatively constant throughout the trial (average beta value of first 28 frames = 0.78) but increased significantly for the last two stimulus frames (average beta value of 29^th^ frame = 1.01, *t*(29) = 5.39, *p* = 0.000009; average beta value of 30^th^ frame = 1.1, *t*(29) = 4.27, *p* = 0.0002; Fig. 4A). Thus, there was a strong recency effect^28,29^ such that the last two frames influenced the decision more than the rest of the frames.

We further computed the ideal information usage for each subject by running the reverse correlation on the stimulus rather than on the response (Fig. 4A). The ideal information usage was higher than the actual. Indeed, a repeated measures ANOVA with factors usage type (actual vs. optimal) and frame showed significant main effects of usage type (F(1,29) = 87.43, *p* < 0.001) and frame (F(24.18,701.12) = 2.29, *p* < 0.001, Huynh-Feldt correction used), as well as a significant interaction between them (F(14.07,408) = 5.24, *p* < 0.001, Huynh-Feldt correction used). The difference between actual and optimal information usage was significant for each of the first 28 frames (all *p*’s < 0.0011; *p* values remain significant after Bonferroni correction for 30 multiple comparisons) but not for the last two frames (29^th^ frame: *t*(29) = −1.3, *p* = 0.2; 30^th^ frame: *t*(29) = 1.23, *p* = 0.23, both *p* values reported before correction).

Critically, we tested whether the temporal information usage varies with the type of cue (Fig. 4B). We computed separately the temporal information usage for predictive/neutral pre and post cues and performed a 2 (cue time; pre/post) × 2 (cue type; predictive/neutral) × 30 (frame number) repeated measures ANOVA. We found no significant effects related to cue time. Specifically, there was no main effect of cue time (F(1,29) = 0.98, *p* = 0.33), interaction between cue time and either cue type (F(1,29) = 0.14, *p* = 0.72) or frame (F(29,841) = 1.19, *p* = 0.23, Huynh-Feldt correction used), or interaction between cue time, cue type, and frame (F(29, 841) = 1.29, *p* = 0.14). Thus, pre and post cues resulted in the same pattern of temporal information usage.

Finally, we tested whether we can detect the signature of decision criterion shift, namely lower usage for predictive compared to neutral cues (Fig. 1E, left). Given that we found no difference between pre and post cues, we combined them together and averaged across the usage over the 30 frames. We found that the information usage was indeed higher for neutral compared to predictive cues (1.42 vs. 1.31, *t*(29) = 3.09, *p* = 0.004), thus confirming that our data are consistent with a decision criterion shift.

### Pre and post cues have the same effect on feature-based information usage

**Figure 5:**
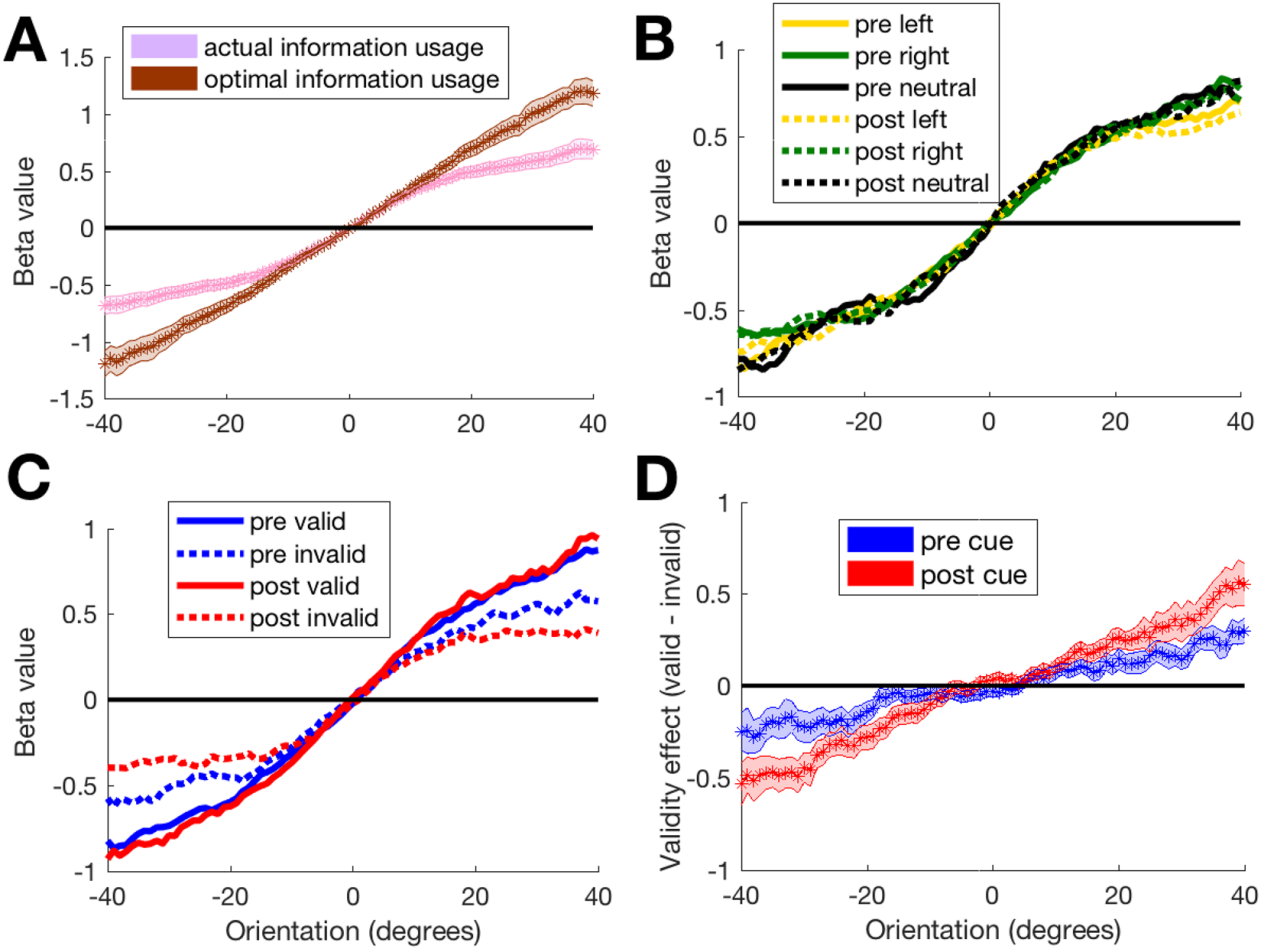
Feature-based information usage. (**A**) The pattern of stimulus information usage across all trials. The graph shows the strength with which each stimulus orientation predicts a “right” (i.e., clockwise) response. The line of optimal usage shows that subjects underweighted the extreme stimulus orientations. (**B**) Feature information usage for each cue identity (left, right, and neutral) did not differ by cue time (pre vs. post). (**C**) The information usage differs between valid and invalid cues. (**D**) The difference between valid and invalid trials is larger for post compared to pre cues. In all panels, 0° indicates vertical orientation and negative (positive) angles indicate counterclockwise (clockwise) deviations from vertical that correspond to left (right) choices. Shaded areas show S.E.M.

The pre and post cues had equivalent effects on the temporal usage of information but it is possible that they induced different feature-based information usage. To understand how subjects used the different feature orientations, as with the temporal usage analyses, we first combined all trials together and examined how different orientations predicted subjects’ choices. Not surprisingly, right orientations predicted right choices, and this relationship was stronger for more extreme orientations, while the opposite was true for left orientations (resulting in positive slope of beta values as a function of orientation, *t*(29) = 9.9, *p* = 8.2 * 10^−11^, Fig. 5A).

When compared to the ideal information usage, orientations close to vertical (defined here as 0° orientation) were weighted optimally but orientations away from vertical were underweighted. A repeated measures ANOVA with factors usage type (actual vs. optimal) and orientation showed a significant main effect of orientation (F(80,2320) = 110.54, *p* < 0.001) and interaction between usage type (actual vs. optimal) and orientation (F(80,2320) = 50.42, *p* < 0.001). Indeed, the actual and optimal curves were significantly different for orientations smaller than −12° and larger than 15° (all *p*’s < 0.05 after Bonferroni correction for 81 comparisons) but not different for orientations between −9° and 9° (all *p*’s > 0.05 even before correction). A similar tendency to down-weight extreme stimuli called “robust averaging” has been observed before in a variety of paradigms^18–21^.

Critically, we tested whether the feature-based information usage varied between pre and post cues (Fig. 5B). We computed separately the feature-based information usage for left/right/neutral pre and post cues and performed a 2 (cue time; pre/post) × 3 (cue identity; left/right/neutral) × 81 (orientation) repeated measures ANOVA. As with temporal information usage, we found no significant effects related to cue time. Specifically, there was no main effect of cue time (F(1,29) = 0.003, *p* = 0.96), interaction between cue time and either cue type (F(1.31,37.92) = 0.83, *p* = 0.4, Huynh-Feldt correction used) or orientation (F(80,2320) = 0.96, *p* = 0.57), or interaction between cue time, cue type, and orientation (F(160,4640) = 0.93, *p* = 0.73). Thus, pre and post cues resulted in the same pattern of feature-based information usage.

Just as with temporal information usage, we tested whether we can detect the signature of decision criterion shift, namely under-weighting of cue-incongruent information (Fig. 1E, right). Indeed, there was a significant interaction between cue identity and orientation (F(160,4640) = 1.452, *p* < 0.001). To highlight the use of cue-congruent and cue-incongruent information, we plotted information usage for valid and invalid cues (Fig. 5C). Invalid cues led to flatter curves (pre cues: *t*(29) = 3.85, *p* = 0.0006, post cues: *t*(29) = 5.4, *p* = 0.000008), indicating the presence of under-weighting of cue-incongruent information. The difference between valid and invalid trials was higher for post cues (as indicated by a steeper slope of the validity effect on orientation, *t*(29) = 4.47, *p* = 0.0001; Fig. 5D), which mirrors the larger post cue effects on criterion *c*.

**Figure 6:**
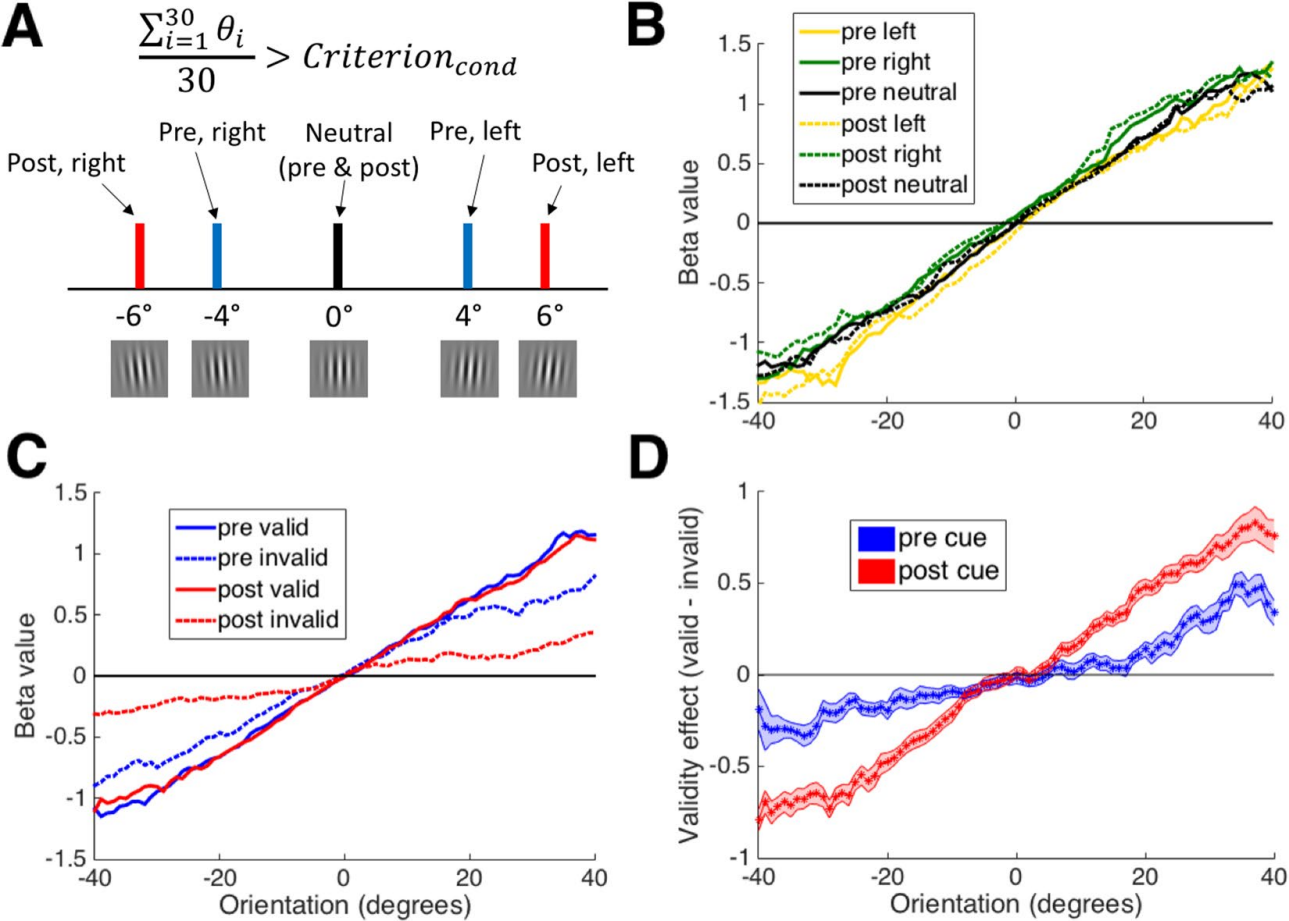
Modeling results. (**A**) Depiction of the model. The model gave responses based on how the average of the 30 Gabor orientations θ_i_ compared to condition-specific criteria. The criteria values used (+/−6° for post cues, +/−4° for pre cues, and 0° for neutral cues) are presented graphically together with a Gabor patch of that orientation. (**B**–**D**) As in Fig. 5B–D, we plot feature information usage for left/right/neutral cues (**B**), for valid and invalid cues (**C**), and for the difference between valid and invalid cues (**D**). The model reproduces the qualitative effects from Fig. 5B–D. In all panels, 0° indicates vertical orientation and negative (positive) angles indicate counterclockwise (clockwise) deviations from vertical that correspond to left (right) choices. Shaded areas show S.E.M.

Finally, we tested the extent to which a simple decision criterion adjustment can produce the effects in Fig. 5C,D. Similar to our simulations in Fig. 1C–E, we created a simple model in which the cues shifted the decision criterion with post cues inducing a larger shift (criteria for pre cues were set at +/−4°; criteria for post cues were set at +/−6°, criterion for neutral conditions was set at 0°; Fig. 6A). Unlike the simulations in Fig. 1C–E, we ran the model on the same trials that subjects experienced. Note that the model does not include mechanisms for down-weighting extreme stimuli. This simple model reproduced the effects of cue identity (left, right, and neutral cues; Fig. 6B; notice the lack of sigmoid shape in the curves due to the equal weighting of all features) and of cue validity (valid and invalid cues; Fig. 6C,D). These modeling results show that the effects of both pre and post cues can indeed be accounted for by a decision criterion shift independent of any effects on the sensory signal.

## Discussion

We set to determine whether stimulus expectation alters subjects’ perceptual judgments by affecting the sensory signal or the decision criterion. To do so, we compared the effects of pre-stimulus cues (pre cues; can affect both the sensory signal and the decision criterion) and post-stimulus cues (post cues; can only affect the decision criterion). Surprisingly, we found that post cues had a greater influence on subjects’ responses than pre cues. More importantly, we observed a substantial inter-subject correlation between the criterion effects of pre and post cues, indicating that the two cues acted via similar mechanisms. Further, pre and post cues did not differ in their effects on stimulus sensitivity, temporal or feature-based information usage. These results suggest that, at least in our experimental design, stimulus expectation affected subjects’ choices by altering the decision criterion rather than the sensory signal.

Our findings provide a strong challenge to claims by Balakrishnan and colleagues who claimed that decision criteria do not shift and used this claim to attack Signal Detection Theory^30,31^. Instead, these authors favor sequential sampling models in which the decision criteria are fixed and change in parameters such as the starting point of the accumulation and drift rate lead to biased responding^32^. However, our results demonstrate that post cues presented significantly after the offset of the visual stimuli can bias the pattern of responses in ways that appear equivalent to pre cues. These findings cannot be accommodated by assuming stable decision criteria since post cues cannot change the starting point of the accumulation or induce a drift rate bias. Therefore, our results demonstrate that, contrary to Balakrishnan’s claims, decision criteria do shift as a result of expectation manipulations.

### Previous demonstrations of sensory signal effects of expectation cues

Despite the apparent lack of sensory signal change in our experiment, it can be argued that several previous studies have shown such an effect. (Note that in most of these studies, the authors did not claim to distinguish between sensory signal and decision criterion.) In the first set of studies, expectation appeared to enhance stimulus sensitivity (*d’*). For example, expecting the stimulus category (e.g., car vs. cat) allowed subjects to detect the stimulus location with greater accuracy^33^. Similarly, valid cues led to more accurate categorization of degraded objects^34^. However, while the cues in these studies consisted of probabilistic information, these cues also provided information about feature relevance. By giving information about the relevance of different features, these studies engaged feature-based attention^35^. Thus, it appears that previous studies showing expectation cues altering stimulus sensitivity did so by inducing attentional changes.

In a second set of studies, expectation did not affect stimulus sensitivity (*d’*) but altered how different stimulus features were used in the decision. For example, expecting a particular orientation enhanced visual sensitivity to off-channel orientations^16^. Similar effects of enhanced off-channel selectivity have been observed in attentional manipulations of feature search^36,37^ suggesting that the expectation cue may have also altered subjects’ feature-based attentional strategy. Another study used a detection task that made attentional changes unlikely and still found a change of sensitivity induced by expectation cues^17^. Even though it is tempting to interpret this result as evidence for expectation altering the sensory signal, such interpretation would be premature. Indeed, the authors modeled their results as contrast gain, which can result from either a change in sensory signal or decisional processes. Thus, expectation-related shifts of stimulus feature usage could be due to decision criterion effects (see also Fig. 1E) and should not be automatically interpreted as evidence for sensory signal change.

### Neural effects of expectation in sensory cortex

A wealth of studies has demonstrated that stimulus expectation leads to changes in early sensory cortex^3,38–48^. It is possible that some of these studies induced a change in strategy thus engaging attention processes. Nevertheless, it is unlikely that all of the above studies can be explained in this manner. Instead, it appears that pure stimulus expectation genuinely affects activity in sensory cortex.

How can we reconcile the neural effects in sensory cortex (which appear to suggest altered sensory processing) and the lack of sensory signal effects in our study? One possibility is that the top-down expectation signals seen in early sensory cortex do not causally contribute to signal processing but simply reflect feedback signals regarding the choice formed in higher-level cortex^11^. Another possibility is that higher-level cortex sends top-down signals to enable sensory cortex to process the stimulus more efficiently^47,49^ but without altering the extracted sensory signal itself ^50^. This latter proposal allows for meaningful neural changes that do not, however, result in differences in the nature of the sensory signal.

Regardless of the exact function of the neural effects in sensory cortex, we agree with Mostert, Kok, and de Lange who argue that “merely recording from sensory areas may be insufficient to disentangle sensory processing from decision-related activity”^51^. As these authors point out, perceptual decision making is the result of complex, reciprocal interactions between many brain regions, and therefore it is difficult (or, perhaps, impossible) to isolate brain activity anywhere in the brain as reflecting the pure sensory signal independent of any top-down decisional influences. Therefore, we believe that unambiguously establishing that expectations affect the sensory signal needs to involve behavioral demonstrations and cannot be purely based on neural activity.

## Conclusion

Our study demonstrated that the effects of pre-stimulus expectation on subjects’ choices can be explained by a change in the decision criterion with no change in the sensory signal itself. Indeed, our model in which expectations only affected the decision criterion captured the effects of both the pre- and post-stimulus cues. Further, in all of our analyses, pre- and post-stimulus cues had equivalent effects. We propose that future studies adopt our pre/post cue design in order to isolate any putative effects of expectation on the sensory signal.

## Methods

### Subjects

Thirty healthy subjects (18 to 24 years old, 13 females) with normal or corrected-to-normal vision participated in the study. All subjects gave written informed consent. The research was approved by the Institutional Review Board of Georgia Institute of Technology. All procedures were performed in accordance with the institutional guidelines. The experiment took approximately one hour to complete and participants were compensated at the rate of $10/hour.

### Task

Subjects indicated whether the overall orientation of a series of Gabor patches was tilted clockwise or counterclockwise from vertical. The Gabor patches were of 100% contrast and appeared within an annulus subtending 1° to 5° at the center of the screen against gray background. Each trial consisted of consecutively presented pre cue period (500 ms), first fixation period (500 ms), stimuli period (500 ms), second fixation period (500 ms), post cue period (500 ms), and untimed response period (Fig. 2A). During the stimulus period, 30 Gabor patches of different orientations were presented for one frame each (one frame = 16.7 ms; Fig. 2B).

The Gabor orientations were drawn randomly from a normal distribution with a standard deviation of 22.5°. The mean of the distribution was determined separately for each subject using a staircasing procedure (average mean = 7.49°, SD = 3.85°). Thus, on average, clockwise/counterclockwise orientations were drawn from a distribution with a mean of +/−7.49° from vertical. The distributions were truncated so that only orientations between −45° and 45° from vertical were shown.

Subjects completed blocks of pre cue and post cue trials. In the pre (post) cue blocks, only the pre (post) cue period contained relevant cues, while the post (pre) cue period contained the same, non-informative symbol “−”. This symbol was presented in order to make the pre and post cue trials as similar as possible to each other.

The relevant cues were either predictive or neutral. The predictive cues indicated that the stimulus is likely to have an overall orientation that is more likely to be left of vertical (i.e., counterclockwise) or right of vertical (i.e., clockwise). These two possibilities were signaled by the symbols “ < ” and “ > ”, respectively. The predictive cues were valid on 66.67% of the trials. The neutral cues were not predictive of the stimulus orientation and consisted of the symbol “|”. Neutral cues were presented on 25% of the trials.

Subjects were fully informed about all of these properties of the cues and were encouraged to take full advantage of the information provided by the cues in their perceptual decisions. In addition, to help subjects use the cues, we provided them with trial-by-trial feedback.

### Procedures

Subjects were first provided with training in which the difficulty of the task was gradually increased by decreasing the offset of the mean of the distribution from which orientations were drawn. At the end of the training, subjects engaged in a staicasing block to establish the individually-determined offset that controls the difficulty of the task. The staircasing block employed an adaptive 2-down-1-up method with decreasing step sizes. The block ended after ten reversals and the threshold was defined as the average of the offset values at the last six reversals. This threshold offset was used in the rest of the experiment.

The main experiment consisted of 480 trials organized in four runs, each consisting of four blocks of 30 trials. Subjects were given a 15-second break between blocks and untimed breaks between runs. For half of the subjects the odd numbered blocks from each run were pre cue blocks, while the even numbered blocks were post cue blocks. For the other half of the subjects, this order was reversed.

### Apparatus

All visual stimuli were created in MATLAB (MathWorks) using Psychtoolbox 3^52^. The visual stimuli were presented on Mac built-in display (1920 × 1080 resolution, 60 Hz refresh rate). Subjects performed the task in a dark room. They were positioned 60 cm away from the screen.

### Analyses

To determine observers’ performance on the task, we computed the Signal Detection Theory measures *d’* (a measure of stimulus sensitivity) and *c* (a measure of response criterion) by calculating the hit rate (HR) and false alarm rate (FAR). Then *d’* and *c* were computed using the following formulas:

1 $$d\mbox{'}=\,{\Phi }^{-1}(HR)-\,{\Phi }^{-1}(FAR)$$

and

2 $$c=-0.5\,\ast \,({{\rm{\Phi }}}^{-1}(HR)+\,{{\rm{\Phi }}}^{-1}(FAR))$$

where *Φ* ^−1^ is the inverse of the cumulative standard normal distribution that transforms HR and FAR into *z*-scores. HR and FAR were defined by treating the right orientation as the target. Therefore, negative *c* values indicate a bias for responding “right,” while positive *c* values indicate a bias for responding “left”.

We computed the optimal *c* value for each condition as the value that maximizes the percent of correct responses. The optimal *c* was derived separately for left and right cues using the formula $\frac{\mathrm{log}(a)}{{d}^{\mbox{'}}}$ where *a* is the odds of presenting left vs. right stimulus (the 66.67% cue validity meant that *a* was 2 for left cues and $\frac{1}{2}$ for right cues). Then, to compute the optimal criterion shift, we subtracted the computed values for left and right cues.

Statistical tests were conducted using t-tests (one-sample and paired) and repeated measures ANOVAs. In cases in which the Mauchly’s test of sphericity was violated, we report the ANOVA results using the Huynh-Feldt correction and note it explicitly in the text.

To derive the maximum correlation between the criterion effects of the pre and post cues, we estimated the reliability of each effect. To do so, we re-computed each effect based only on the odd vs. even trials. However, since this analysis only uses one half of the available trials, it may underestimate the reliability of the measured effects when all trials are used. To correct for this issue, we applied the Spearman-Brown formula^27^ that computes the expected reliability of the full test based on the reliability of one half of it:

3 $${r}_{full}=\frac{2\,\ast \,{r}_{half}}{1+{r}_{half}}$$

The reliabilities for pre and post cue effects were 0.65 and 0.76 but after applying the Spearman-Brown correction, they were estimated as 0.79 and 0.86. We then computed the maximum predicted correlation between the pre and post cue criterion effects by applying the correction for attenuation^27^:

4 $${r}_{maximum}=\sqrt{{r}_{measure1}\,\ast \,{r}_{measure2}}$$

where *r*_*maximum*_ is the maximum correlation between measures 1 and 2, and *r*_*measureX*_ is the reliability of measure X (for X = 1, 2). Finally, we compared *r*_*maximum*_ with the correlation that we actually observed *r*_*observed*_.

Temporal reverse correlation analyses were performed on the raw orientation values in radians. Vertical orientation was coded as 0° and clockwise (counterclockwise) offsets from vertical were coded as positive (negative) values. The orientation values were entered into a logistic regression that predicted subjects’ choices. Optimal information usage was determined by performing the same logistic regression on the stimulus identity instead. The logistic regression for each condition was performed separately on the orientations from each of the thirty frames.

The feature-based reverse correlation analyses were performed via a logistic regression on the orientation values in degrees. For each orientation (i.e., feature) *θ* between −40° and +40° (whole numbers only), we calculated the number of Gabor patches with orientation in the interval [*θ* − 5°, *θ* + 5°]. We obtained one such number per trial, and these numbers were used as predictors in logistic regressions performed for each orientation angle. Thus, for each condition, we performed 81 regressions. In three cases (out of a total of 24,300 regressions performed), subjects’ responses could be predicted perfectly resulting in extreme beta values (*θ* = −38° for the neutral pre cues condition in subject 15, and *θ* = 32° and 33° for the neutral post cues condition in subject 3). The beta values from these regressions were replaced with the weighted beta values derived from the regressions for the neighboring *θ* values for the same subject.

### Simulations

We simulated three different possible effects of expectation cues in order to demonstrate that reverse correlation analyses are sensitive to such effects. In three analyses, we simulated a change in early but not late sensory signal (Fig. 1C), a change in ambiguous but not unambiguous sensory signals (Fig. 1D), and a change in the decision criterion (Fig. 1E). In each case, we simulated 1 million trials with each type of cue (left, right, and neutral) using the same parameters as in the main experiment.

In the first simulation (change in early signal), the information from the first six frames was weighted 8 − *i* times more than the rest of the frames, where *i* is the frame number. Importantly, this higher weighting was applied only for cue-congruent orientations. In the second simulation (change in ambiguous signals), orientations close to vertical were altered in the direction of the cue. For right (left) cues, an orientation of *θ* was transformed to $\theta +(-)\,240\,\ast \,\varphi (\theta |\mu ,{\sigma }^{2})$, where μ = 0, σ = 6, and $\varphi (\theta |\mu ,{\sigma }^{2})$ is the probability density at *θ* of a Normal distribution with a mean of μ and standard deviation of σ. In the third simulation (change in decision criterion), the decision criterion was set to 4° (−4°) for left (right) cues. The decision was always made by comparing the average of the 30 orientations to the criterion (which was kept at 0° in the first two simulations, as well as the neutral cue condition of the third simulation). In all cases, the simulation parameters were chosen so that they produced similar average amount of bias as in the real data.

### Modeling

We attempted to reproduce the feature-based reverse correlation effects by creating a simple model of the cuing effects. In our model, “right” (i.e., clockwise) choices were made if the average of the 30 orientations *θ*_*i*_ presented on a single trial exceeded a condition-specific criterion. Formally, “right” responses were given when:

5 $$\frac{{\sum }_{i=1}^{30}{\theta }_{i}\,}{30} > Criterio{n}_{cond}$$

*Criterion*_*cond*_ was set to 4°/6° for pre/post left cues (the positive values ensure that “left” responses are more likely), −4°/−6° for pre/post right cues (the negative values ensure that “right” responses are more likely), and 0° for both pre and post neutral cues (ensuring unbiased responses). Even though in reality these criteria must have varied across subjects, we used the same criteria values for all subjects to avoid overfitting and since the model was designed as a simple proof-of-concept.

Rather than creating idealized predictions of the model (as in Fig. 1C–E), which would obscure the noise inherent in real data, we ran the model directly on the actual stimuli experienced by the subjects. We performed the exact same logistic regressions as in our feature-based analyses. Just as with those analyses, a few logistic regressions resulted in perfect predictions and therefore extreme beta values. To correct for this, we excluded the data from 4 subjects where perfect predictions occurred for too many regressions and replaced individual extreme beta values for non-excluded subjects with the weighted beta values derived from the regressions for the neighboring *θ* values (using the same procedure as for the real data; overall, 10 such substitutions we made).

### Data and code availability

All data and codes for the analyses, including the modeling and simulations, are freely available online at: https://github.com/DobyRahnev/pre_post_cues.
